# Identification of DNA Methylation Differences in Pituitary Tissues of Sichuan White Geese Using Whole-Genome Bisulfite Sequencing (WGBS)

**DOI:** 10.3390/biology14020154

**Published:** 2025-02-03

**Authors:** Lin Ma, Xianzhi Zhao, Guoda A, Tongtong Song, Meng Wu, Zhihao Yan, Min Xiao, Wenbo Jiang, Yixiao Gao, Haiwei Wang, Zhuping Chen, Keshan Zhang, Jiajia Xue, Yi Luo, Chao Wang, Youhui Xie, Ying Chen, Guangliang Gao, Qigui Wang

**Affiliations:** 1 Chongqing Engineering Research Center of Goose Genetic Improvement, Institute of Poultry Science, Chongqing Academy of Animal Science, Chongqing 402460, China; 17783415478@163.com (L.M.); zhaoxianzhi2002@163.com (X.Z.); wahwe@163.com (H.W.); 2019102008@stu.sicau.edu.cn (Z.C.); zhangkshlk1988@163.com (K.Z.); xuejiajia25@163.com (J.X.); homleestar@163.com (Y.L.); wangccq@foxmail.com (C.W.); xyh917111@163.com (Y.X.); chenying.cq@163.com (Y.C.); 2 College of Animal Sciences and Technology, Northeast Agricultural University, Harbin 150030, China; aguoda02@gmail.com (G.A.); 13191668352@163.com (T.S.); 3 College of Animal Science and Technology, Southwest University, Chongqing 402460, China; wumeng342@outlook.com (M.W.); mondragondeiontonio@gmail.com (Z.Y.); 19562241901@163.com (M.X.); j24130@email.swu.edu.cn (W.J.); 17793151770@163.com (Y.G.)

**Keywords:** Sichuan white geese, egg-laying traits, pituitary, whole-genome bisulfite sequencing (WGBS)

## Abstract

To explore the impact of epigenetic modifications on egg-laying traits in geese, we employed genome-wide bisulfite sequencing (WGBS) to analyze DNA methylation patterns in pituitary tissues of high-(HYP) and low-yield (LYP) Sichuan White geese. We achieved high-quality sequencing data (mean 19.09 Gb raw reads, 15.49 Gb clean reads, 79.1% unique mapping rate) with a bisulfite conversion efficiency of 99.88%. Comparative analysis revealed 2394 differentially methylated regions (DMRs) and 422 differentially methylated genes (DMGs) between HYP and LYP groups. We identified five key differentially methylated candidate genes (*BMPER*, *INHA*, *NMBR*, *NK3R*, and *DSG2*) linked to egg-laying traits in Sichuan White geese. Integrated GO and KEGG enrichment analysis conducted to explore the role of regulatory networks of epigenetic modification on egg-laying traits in Sichuan White geese identified multiple metabolic pathways associated with egg-laying traits (promoting egg transport, ovulation, and yolk protein synthesis and secretion), thus providing a basis for subsequent functional verification.

## Simple Summary

High egg production is a significant economic trait in geese. Whole-genome bisulfite sequencing (WGBS), a commonly used technique, facilitates the identification of key differentially methylated candidate genes associated with egg-laying traits by analyzing DNA methylation differences and has been widely applied in studies of epigenetic regulation mechanisms in animals. However, genome-wide studies investigating the association between DNA methylation and egg-laying traits in geese are currently lacking. In this study, we performed DNA methylation profiling on pituitary tissues from six Sichuan White geese in both high-yield (HYP) and low-yield (LYP) groups. The results revealed five differentially methylated candidate genes significantly associated with egg-laying traits in Sichuan White geese, including *BMPER*, *INHA*, *NMBR*, *NK3R*, and *DSG2* genes. This study provides a theoretical basis for further elucidating the epigenetic regulatory mechanisms of egg-laying traits in geese.

## 1. Introduction

China’s goose industry holds a significant position in the global waterfowl sector, possessing over 80% of the world’s goose genetic resources and accounting for more than 90% of global breeding capacity [1,2]. However, surveys of domestic geese in China reveal that Sichuan White geese have an egg production rate of only 48.60% [3]. This rate is 16.51% higher than that of Rhine geese (32.09%) and 0.67% higher than that of Wanxi White geese (41.43%) [4,5], but 1.4% and 16.51% lower than that of Italian White (50.00%) and White Roman geese (85.24%), respectively [6,7]. Thus, improving the egg production of Sichuan White geese remains a key challenge. Egg production in geese is a complex trait influenced by multiple factors, including reproductive hormones (e.g., melatonin (MLT) and progesterone (P4)), breed characteristics, and disease [8,9,10]. Notably, physiological traits of geese, such as a short breeding period, pronounced nesting behavior, and seasonality, often lead to reduced egg production or infertility. This not only increases the cost of breeding geese but also significantly diminishes economic returns, posing a major barrier to the development of the goose breeding industry [11,12,13].

To date, researchers have tackled the challenge of the poultry-laying rate through studies of nutritional regulation, traditional mating breeding, and molecular genetics. Nutritional regulation is a widely adopted strategy. For instance, Yu et al. demonstrated that a 0.02% taurine diet regulates estradiol via ovarian secretion, enhancing the synthesis, transport, and deposition of yolk precursors, very-low-density lipoprotein, and vitellogenin, thereby promoting follicle development and ovulation in late-laying hens, ultimately improving the laying rate of hens in the late laying period [14]. Similarly, Fan et al. investigated the impact of zinc (Zn) and *Bacillus subtilis* (*B. subtilis*) supplementation on reproductive performance in geese breeders, finding that a combination of 5 × 10^9^ CFU/kg *B. subtilis* and 25 mg or 45 mg/kg Zn significantly improved egg-laying rate, fertility, hatchability, and yolk color [15]. Previous studies show crossbreeding significantly enhances egg production and quality, with parameters like heterosis and combining abilities underscoring its key role in poultry genetic improvement [16].

In addition, the molecular genetics level is also a research focus, with many researchers using gene expression regulation, genetic marker selection, and other means to explore the factors affecting poultry egg production. For example, a quantitative analysis of gene expression in ovarian tissue by Chen et al. showed that the expression of lipid synthesis genes *HMGR*, *Apo-B2*, and *SREBP-2* was regulated by egg-laying-related hormones [17]. Sun et al. identified two candidate genes, *NDUFAB1* and *GABRA1*, that may be linked to egg-laying performance in chickens through comparative ovarian transcriptomics [18]. Furthermore, Han et al. employed DNA methylation analysis to identify methylation sites associated with inbreeding depression in chicken reproduction through WGBS of hypothalamus and ovary tissues, uncovering 5948 and 4593 differentially methylated regions (DMRs), and two potential biomarkers, *SRD5A1* and *CDC27*, linked to reproduction-related pathways [19].

Prior studies have shown that DNA methylation exhibits highly specific and stable heritable characteristics, with methylation modifications predominantly occurring at cytosine-guanine (CpG) dinucleotides. CpG islands are typically located in gene promoter regions. For example, Wang et al. generated a DNA methylation map of muscle tissue from Chinese Chenghua pigs (CHP) and found that methylation was largely concentrated at CpG sites, averaging 51.39%, while non-CpG sites exhibited only 1.66% methylation [20].

Currently, whole-genome bisulfite sequencing (WGBS) provides a comprehensive view of CpG site methylation status across the genome, offering high-resolution methylation maps essential for understanding complex biological processes and gene regulatory networks. Yuan et al. have begun to explore WGBS in pig pituitary tissues, demonstrating that DNA methylation disruption can delay initial estrus. Functional analysis revealed the enrichment of differentially methylated genes in signaling pathways related to gonadotropin (Gn) release by the pituitary [21]. However, few studies have investigated the epigenetic mechanisms of WGBS in goose pituitary tissue. Most studies have focused on WGBS in ovarian and hypothalamic tissues, providing evidence that DNA methylation regulates these tissues. Liu et al., using WGBS to study the hypothalamus of Magang geese across three reproductive stages, found that methylation of the serotonin metabolic pathway gene *5-HIAA* is associated with the regulation of egg-laying traits [22]. Comparing ovarian DNA methylation profiles between high- and low-yield Huyang geese, Zhang et al. found ten differentially methylated genes (DEGs), including *BMP7*, *BMPR1B*, *CTNNB1*, *FST*, and *FSH*, likely involved in reproduction [23]. Yao et al. also explored DNA methylation patterns in Huyang goose ovaries, revealing genetic mechanisms underlying high fertility [24]. Furthermore, Zhang et al. analyzed genome-wide DNA methylation in hypothalamus and ovarian tissues across five stages of FM, identifying five genes that regulate the expression of egg-laying traits during FM, potentially serving as biomarkers for regulating these processes [25].

Collectively, these findings suggest that DNA methylation participates in regulating animal reproduction and egg production, offering a new perspective for optimizing breeding strategies and enhancing egg production rates. Here, we collected pituitary tissues from high- and low-yielding geese and performed WGBS to investigate their DNA methylation profiles. Our objectives were to identify candidate genes and metabolic pathways associated with high yield in female geese, further elucidate pituitary epigenetics in geese, and provide a theoretical framework for breeding high-yielding female geese and developing superior germplasm resources.

## 2. Materials and Methods

### 2.1. Collection of Animal Samples

The experiment was reviewed and approved by the Experimental Animal Ethics Committee of Chongqing Academy of Animal Science. This study was conducted at the Poultry Research Base of Chongqing Academy of Animal Science (Rongchang Anfu, Chongqing, China). All Sichuan White geese were fed the same standardized feed in a single cage and had free access to food and water. The experiment lasted from September 2017 to February 2018, and six 45-week-old female Sichuan White geese from the same batch were randomly selected. According to their egg production, they were divided into a high-yielding group (HYP, *n* = 3, 55.76 ± 1.61) and a low-yielding group (LYP, *n* = 3, 31.29 ± 1.23). The experimental design began with an observation group of 230 female geese selected based on egg production performance. From this initial group, 34 geese were chosen according to the number of eggs produced, consisting of 17 high-yielding and 17 low-yielding individuals. The current experiment utilized six female geese selected from those 34, with 3 geese from the high-yielding group and 3 from the low-yielding group. The six geese were not full or half-siblings and were derived from distinct, unrelated family lineages. Each goose was slaughtered and dissected 2 h before laying by the method of egg supporting. The head of the goose was dissected with pliers, and the pituitary tissue was extracted, frozen in liquid nitrogen, and stored in an ultra-low temperature refrigerator at −80 °C for future use.

### 2.2. Pituitary Tissue DNA Extraction and Whole Genome Methylation Sequencing Library Construction

After the pituitary tissue was ground into powder, 25 mg of pituitary tissue was purified by silica gel membrane adsorption column QIAamp DNA mini-51304 Kit (Qiagen, Hilden, Germany) combined with a unique Qiagen buffer. DNA was extracted by cleavage, protease K degradation, and elution. A NanoDrop 2000 spectrophotometer (Thermo Scientific, Waltham, MA, USA) was used to detect the OD260/280 ratio of DNA. DNA samples were generally determined to have 1.8 ≤ OD260/280 ≤ 2.0, and the concentrations were greater than 40 ng/μL. The results of agarose gel electrophoresis showed clear DNA bands and no trailing, and no additional bands were detected, indicating that the DNA samples were not degraded or contaminated, ensuring the high purity and integrity of the DNA samples. Qualified DNA samples were segmented to 200–300 bp using Covaris S220 ultrasonic crusher (Woburn, MA, USA). After end repair, a single adenine was added to the 3′ end of the fragmented DNA, allowing for ligation to sequencing adapters. Bisulfite conversion was conducted using EZ DNA Methylation-Gold™ D5005 Kit (Zymo Research, Orange County, CA, USA) to convert unmethylated cytosine to uracil, while methylated cytosine remained unmodified. PCR amplification was then performed. The quality of the library was assessed using the Agilent 2100 bioanalyzer (Agilent Technologies, Santa Clara, CA, USA), which showed a single peak in the 400–500 bp range, and the peak shape was free of trailing or acromial peaks. The Illumina HiSeq™ 2500 platform (Illumina, San Diego, CA, USA) was used for double-ended 150 bp sequencing with a depth of >30× per sample. The sequencing data from pituitary tissues of Sichuan White geese have been deposited in the NCBI Sequence Read Archive (SRA) under accession IDs SRR8118674, SRR8118672, SRR8118669, SRR8118670, SRR8118667, and SRR8118668.

### 2.3. DNA Methylation Sequencing Analysis

The Trimmomatic (version 0.39) software qualitatively controls the raw sequencing data, removing splicing sequences and low-quality bases (QPhred < 20). Filtered high-quality clean reads were compared with the goose reference genome (version ASM1303099v1) using Bismark (version 0.23.0) software. The bisulfite conversion rate, i.e., the proportion of methylated clean reads to the reference genome, was calculated. The methylation information of cytosine sites was extracted based on the principle of cytosine–thymine conversion. The unmethylated cytosine was converted to thymine, while the methylated cytosine remained unchanged. Cytosine sites were classified into CpG, CHG, and CHH contexts (where H stands for A, T, and C). The binomial distribution test was used to determine methylation status, and cytosine sites with coverage > 4× and false discovery rate (FDR) < 0.05 were identified as methylation sites. Genome-wide methylation levels were analyzed using a 10,000 bp window size with 500 bp overlap, and DNA methylation data were visualized using Viewbs (version 2.0) software. The methylation level was calculated as follows:

$$Methylation\;level\;(\%)=\frac{reads\;mC}{reads\;mC+reads\;umC}$$

mC and umC represent the number of reads of methylated and unmethylated cytosine, respectively.

### 2.4. Identification of Differentially Methylated Regions and Differentially Methylated Genes

DSS (version 2.52.0) software was used to identify differential methylation regions (DMRs) of pituitary tissue in the high-yield and low-yield groups. Areas with *p* < 0.05 and |diffMethy| > 0.2 were considered as significant DMRs. The genes annotated by DMRs are called differentially methylated genes (DMGs).

### 2.5. Functional Enrichment Analysis of Differentially Methylated Genes

Metascape online tools (https://metascape.org/gp/index.html#/main/step1, accessed 1 October 2024) were used to generate histograms of GO DMG enrichment analysis. Items |Log2FC| ≥ 1 and *p* < 0.05 were considered to be significantly enriched. The minimum overlap number for the input gene list was set to 3, and the minimum enrichment factor was set to 1.5. KEGG pathway enrichment analysis was conducted using GENE DENOVO online tools (https://www.omicshare.com/tools/, accessed 15 August 2024). The database version link: https://ftp.ensembl.org/pub/release-109/gtf/homo_sapiens/ (accessed on 27 January 2023).

## 3. Results

### 3.1. Summary of Pituitary DNA Methylation Sequencing Data

As detailed in Table 1, the high-yield group (HYP) and the low-yield group (LYP) yielded an average of 19.55 Gb and 18.63 Gb of raw reads, respectively; after quality filtering, these values were reduced to 15.61 Gb and 15.37 Gb of clean reads, respectively. Furthermore, the rates of unique mapping to the goose genome were 77.93% and 80.27% for the high- and low-yield groups, respectively, indicating that the data obtained were abundant and of high quality. Bisulfite conversion metrics are shown in Table 2. The average bisulfite conversion efficiency was 99.88% across all samples in both the high- and low-yield groups, demonstrating the high quality of the bisulfite sequencing data suitable for subsequent methylation analysis.

### 3.2. Analysis of Sequencing Depth and Sulfite Conversion Efficiency

**Figure 1:**
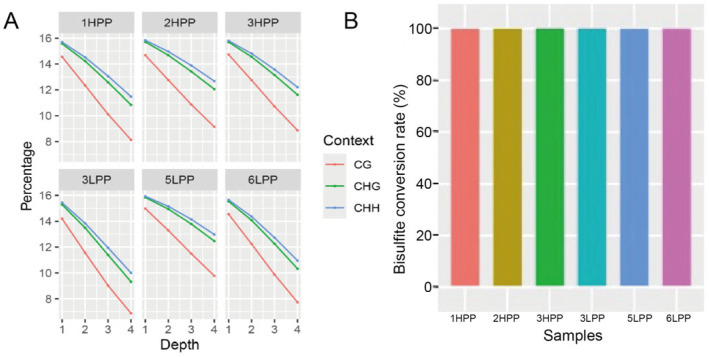
Visualization of pituitary DNA methylation sequencing results. Note: (**A**) Methylation sequencing depth of different samples. Red, green, and blue represent mCpG, mCHG, and mCHH, respectively. (**B**) Bisulfite conversion efficiencies of different samples. Red, yellow, and green represent 1HPP, 2HPP, and 3HPP, respectively; blue, light blue, and pink represent 1LPP, 2LPP, and 3LPP, respectively. 1HPP to 3HPP represent the samples from group HYP, and 4LPP to 6LPP represent the samples from group LYP.

Sequencing depth statistics for whole-genome methylation of pituitary tissue are presented in Figure 1A. These data show that the methylation sequencing depth for each individual sample in both the high-yield (1HPP, 2HPP, and 3HPP) and low-yield (4LPP, 5LPP, and 6LPP) groups was ≥4 at CpG, CHG, and CHH sites, indicating sufficient coverage. Sites with insufficient sequencing depths were removed. These findings suggest that the methylation sequencing was reliable, and the accuracy of subsequent analyses is high. Bisulfite conversion efficiencies are shown in Figure 1B and reveal average conversion rates above 99% across all samples, indicating successful conversion of most cytosine residues and accurate reflection of the true methylation status by the sequencing data.

### 3.3. Analysis of the Methylation Level of Pituitary Tissue Sites

**Figure 2:**
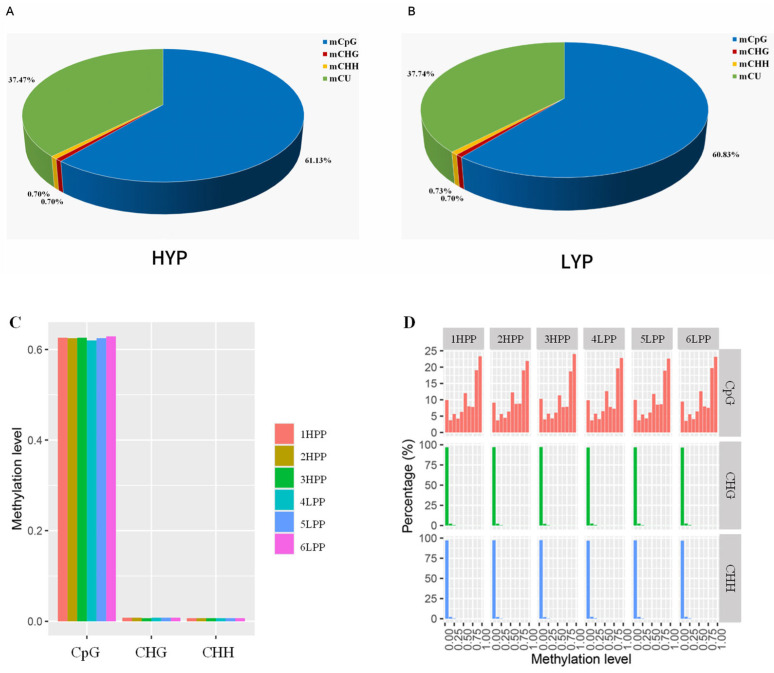
Methylation levels of each methylation type in pituitary tissue. (**A**) Average proportions of DNA methylation types in the hypophyseal tissue of the HYP group, with blue, red, yellow, and green representing mCpG, mCHG, mCHH, and mCU, respectively. (**B**) Average proportions of DNA methylation types in the pituitary tissue of the LYP group, with blue, red, yellow, and green representing mCpG, mCHG, mCHH, and mCU, respectively. (**C**) DNA methylation levels in pituitary tissue, with red, yellow, and green representing samples 1HPP, 2HPP, and 3HPP, respectively, and blue, light blue, and pink representing samples 1LPP, 2LPP, and 3LPP, respectively. (**D**) Proportions of DNA methylation types in pituitary tissue. Red, green, and blue represent CpG, CHG, and CHH, respectively; samples 1HPP to 3HPP are from the HYP group, and samples 4LPP to 6LPP are from the LYP group.

Bismark was used to detect methylation sites in pituitary tissue from high- and low-yielding groups. The results showed that the number of cytosine methylation at CpG sites was more than that at CHG and CHH sites (Table 3). Figure 2A–C illustrate the distribution of DNA methylation types in pituitary tissues from high-yield (HYP) and low-yield (LYP) groups. Methylation proportions exceeded 61% at CpG sites in both HYP and LYP groups, whereas proportions at CHG and CHH sites were below 0.70%, indicating that mCpG is the predominant methylation mode in pituitary tissues of Sichuan White geese. Figure 2D shows the distribution of methylation across different sequence sites (CpG, CHG, and CHH) divided into bins. Each bin shows the number of methylated cytosines in each site. Results indicate that the CpG site had more bins and more methylated cytosines compared to CHG and CHH. These results demonstrate that methylation in pituitary tissues of Sichuan White geese is primarily concentrated at CpG sites in both high- and low-yield groups.

### 3.4. Methylation Characteristics of Differential Methylation Regions

**Figure 3:**
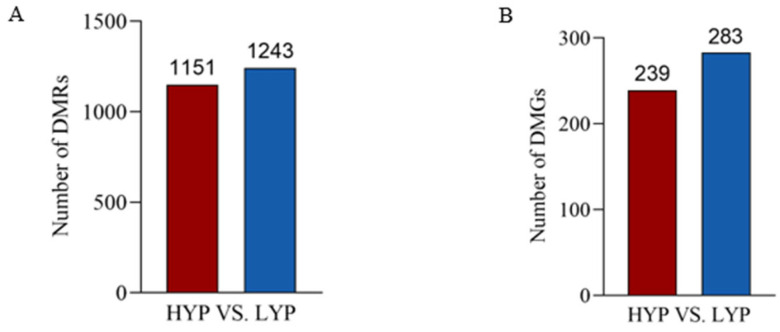
(**A**) Number of DMRs in pituitary tissue. Columns displaying hypermethylated DMRs are colored red, and those displaying hypomethylated DMRs are colored blue. (**B**) Number of DMGs in pituitary tissue. Columns displaying hypermethylated DMGs are colored red, and those displaying hypo-methylated DMGs are colored blue.

DSS software identified a total of 2394 differentially methylated regions (DMRs) in pituitary tissue between the high- and low-yield groups. The DMRs were predominantly hypomethylated; specifically, we identified 1243 hypomethylated DMRs and 51 hypermethylated DMRs (Figure 3A).

### 3.5. Identification of Differentially Methylated Genes (DMGs)

**Figure 4:**
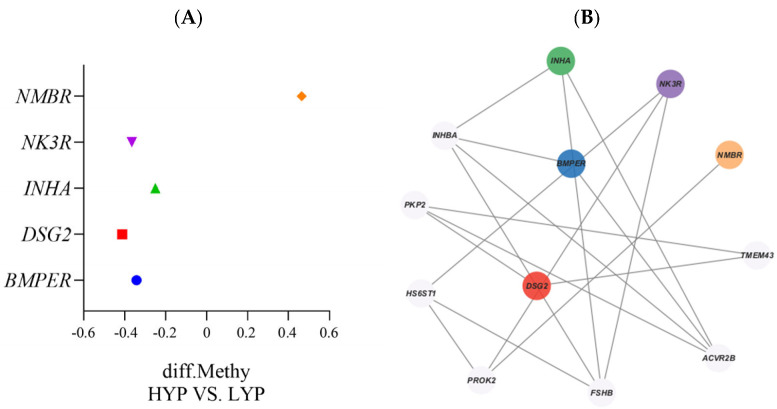
Differential methylation genes (DMGs). (**A**) Number of DMGs in pituitary tissue. (**B**) Network interaction map of traits related to egg laying in pituitary tissue. The lines represent correlations between interacting genes.

We identified 523 differentially methylated genes (DMGs) within the 2394 differentially methylated regions, with 283 exhibiting hypomethylation and 239 exhibiting hypermethylation, indicating a prevalence of DMGs with hypomethylation (Figure 3B and Figure 4A). Figure 4B illustrates the network interaction relationship between DMGs and genes related to egg-laying traits. Three DMGs (*TACR3*, *INHA*, and *BMPER*) are key genes and were hypomethylated.

### 3.6. Functional Enrichment Analysis of Differential Methylated Genes (DMGs)

**Figure 5:**
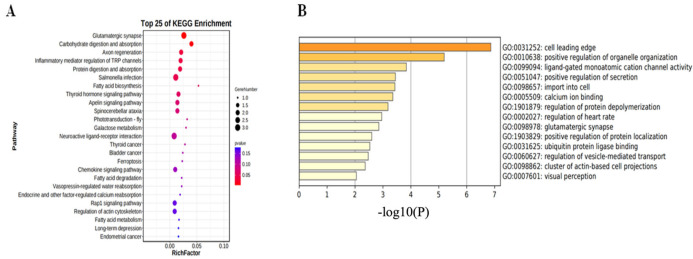
GO and KEGG enrichment. (**A**) GO enrichment analysis of differentially methylated genes (DMGs). (**B**) KEGG enrichment analysis of differentially methylated genes (DMGs).

GO functional enrichment analysis, as shown in Figure 5A, revealed that differentially methylated genes (DMGs) in Sichuan White geese were primarily enriched in pathways related to the positive regulation of secretion (GO:0051047, 5/313, −LogP = −3.44), regulation of vesicle-mediated transport (GO:0060627, 5/519, −LogP = −2.47), actin-based cell projection organization (GO:0098862, 3/168, −LogP = −2.36), positive regulation of organelle organization (GO:0010638, 8/511, −LogP = −5.19), and cellular import (GO:0098657, 7/690, −LogP = −3.43). Notably, these GO terms (GO:0060627, GO:0051047, GO:0098657) were associated with egg-laying traits in geese.

KEGG pathway analysis, detailed in Figure 5B, highlighted the glutamatergic synapse signaling pathway as exhibiting the most significant enrichment. Additionally, the enrichment of the neuroactive ligand–receptor interaction and apelin signaling pathways suggests a potential link between DMGs and egg-laying traits.

## 4. Discussion

In this study, we performed WGBS of pituitary tissue samples from HYP and LYP Sichuan White geese. We obtained 19.55 and 18.63 Gb of raw reads and 15.61 and 15.37 Gb of clean reads, respectively. The mapping rates of 77.93% and 80.27% indicate high sequencing quality, providing a reliable foundation for subsequent analyses. We also found that DNA methylation at CpG sites exceeded 61% in both HYP and LYP groups but remained below 0.70% at CHG and CHH sites, suggesting that mCpG is the dominant DNA methylation pattern in Sichuan White goose pituitary tissue. This finding aligns with the mCpG-dominant DNA methylation observed in chicken (Fayoumi and Leghorn) tissues [26]. In addition, our results showed that the number of cytosine methylation at CpG sites was more than that at CHG and CHH sites, confirming the same results in studies on mice, cattle, and Berkshire pig placenta [27,28,29]. The characteristic CpG pattern of DNA methylation arises from the preferential action of DNA methyltransferases (DNMTs) on CpG dinucleotides. Consequently, mCpG is abundant in vertebrate genomes, especially within promoter regions, gene bodies, and repetitive elements, and is essential for gene expression regulation and transcriptional silencing [30,31]. In animal breeding, alterations in DNA methylation patterns are strongly associated with breeding selection, egg-laying traits, and reproductive success. Analyzing methylation patterns in individuals enables the identification of genes and signaling pathways linked to desired traits, which is crucial for breeding and molecular marker development [32].

We identified five DMGs (*BMPER*, *INHA*, *NMBR*, *NK3R*, and *DSG2*) associated with egg-laying traits in the pituitary tissues of HYP and LYP Sichuan White geese. *NMBR* exhibited high methylation levels, while the other four genes displayed low methylation. The *INHA* gene, encoding an ovarian-derived hormone, regulates follicle-stimulating hormone (FSH) secretion from the anterior pituitary, maintaining normal FSH levels via feedback mechanisms and influencing follicle development and ovulation [33,34]; for instance, Liu et al. showed that the IGF1, an upstream regulator in 6–8 mm follicular granulosa cells, positively influences *INHA* gene expression, resulting in enhanced ovulation in Zhedong White geese [35]. Similarly, Cui et al. found the highest *INHA* mRNA levels in chicken F5 follicles, suggesting a role for *INHA* in regulating follicle development in egg-laying hens [36]. Furthermore, Chen et al. reported that *INHA* affects follicle development to modulate sexual maturation time [37]. *BMPER*, an important regulator of the *BMP* (TGF-β superfamily) signaling pathway, promotes follicle development, neovascularization, follicle maturation, and ovulation when hypomethylated [38,39]. Currently, there is a lack of research investigating the *BMPER* gene in poultry. Studies on the *BMPER* gene have primarily focused on ruminants. For example, Zhang et al. revealed the regulatory role of *BMPER* on ovarian follicle development in Tibetan sheep, finding that *BMPER* also modulates granulosa cell proliferation and differentiation, thereby affecting estrogen and progesterone synthesis [40,41,42]. Ye et al. demonstrated that the *NK3R* gene, involved in the regulation of steroid hormones like estradiol, exhibits extensive interaction with genes in the pituitary gland during the reproductive cycle. Separately, Wu and Meng et al. found that the *NK3R* gene (*TACR3*) is highly expressed in the chicken hypothalamus and that estrogen (E2) can regulate mRNA expression of *TAC3* and *GnRH* and the secretion of GnRH/LH by dimerizing nuclear estrogen receptor 1 (ESR1), thus regulating ovulation and maintaining the reproductive cycle [43,44,45]. The *DSG2* gene has not been studied in poultry, but it has been studied in cattle and marsupials. Douville and Dudley et al. found that *DSG2* is involved in maintaining endometrial stability. Shemesh later demonstrated that the uterus responds to LH, creating a favorable environment for egg transport and fertilization post-ovulation [46,47,48]. Thus, in our study, hypomethylation of *BMPER*, *INHA*, *NK3R*, and *DSG2* may promote egg vesicle development and ovulation in the high-yield group, thus increasing egg production in these geese.

The *NMBR* gene remains unstudied in poultry, with limited exploration in two publications focused on pigs. Ma et al. demonstrated in two reports that *NMBR*, a receptor for Neuromedin B (*NMB*), binds to *NMB* and that *NMB* may regulate GnRH release through modulation of *NMBR* secretion. Furthermore, *NMBR* expression in the myometrium contributes to uterine smooth muscle contractility [49,50]. In our study, the hypermethylation of *NMBR* in the low-yield group may cause the *NMB* gene to inhibit GnRH release, affecting LH secretion and ultimately leading to delayed ovulation and reproductive dysregulation, thus reducing egg production. Furthermore, *NMBR* hypermethylation may reduce uterine smooth muscle contractility, resulting in difficulty in egg laying.

Our GO enrichment analysis indicated that differentially methylated genes (DMGs) were enriched in pathways related to the positive regulation of secretion (GO:0051047), regulation of vesicle-mediated transport (GO:0060627), actin-based cell projection organization (GO:0098862), positive regulation of organelle organization (GO:0010638), and cellular import (GO:0098657). Three of these terms are linked to egg-laying traits. Kui et al. demonstrated that cellular import (GO:0098657) is primarily involved in ovarian follicle development [51]. Sun et al. found that regulation of vesicle-mediated transport (GO:0060627) is crucial for eggshell formation; uncalcified eggs immersed in uterine fluid rely on vesicle-mediated transport, particularly to form a robust eggshell [52]. Szeszko et al. showed that positive regulation of secretion (GO:0051047) plays a key role in regulating female reproductive function by affecting the hypothalamic–pituitary–gonadal axis and influencing follicular development and ovulation [53]. Concurrently, our KEGG pathway analysis revealed DMG enrichment in the neuroactive ligand–receptor interaction and apelin signaling pathways (Figure 5), suggesting the roles of these pathways in reproductive activities related to egg production. Tang et al. also demonstrated the importance of these pathways in controlling reproductive activities of the pituitary, testis, and external genitalia [54]. Schilffarth et al. showed that apelin is expressed in ovarian follicles [55]. Furthermore, Kurowska et al. reported that the apelin system participates in blood vessel formation during follicle cell formation and maturation [56]. Combined, our GO and KEGG enrichment analyses suggest that DMGs contribute to egg formation by regulating follicle development and ovulation during reproductive activities, thereby influencing egg production in geese. These findings offer a crucial theoretical framework for further elucidating the molecular mechanisms underlying improved egg-laying traits.

## 5. Conclusions

Our whole-genome bisulfite sequencing (WGBS) of pituitary tissue in Sichuan White geese identified five key differentially methylated genes (*BMPER*, *INHA*, *NMBR*, *NK3R*, *DSG2*) associated with egg-laying traits, providing an epigenetic foundation for future breeding strategies aimed at enhancing egg production.

## Figures and Tables

**Table 1:** Summary of raw data quality control.

Groups	Sample	Raw Reads	RB (Gb)	Clean Reads	CB (Gb)	Clean Ratio (%)	UM (%)
HYP	1HPP	134,558,301	20.18	103,031,684	15.45	76.57	74.60
	2HPP	121,091,719	18.16	101,039,236	15.16	83.44	81.40
	3HPP	135,339,304	20.30	108,062,512	16.21	79.85	77.80
LYP	4LPP	121,693,283	18.25	98,030,977	14.70	80.56	78.50
	5LPP	126,492,354	18.97	103,536,970	15.53	81.85	79.90
	6LPP	124,378,102	18.66	105,818,840	15.87	85.08	82.40

Note: RB: Raw bases; CB: Clean bases; UM: Unique mapping rate; 1HPP to 3HPP represent the samples from group HYP; 4LPP to 6LPP represent the samples from group LYP.

**Table 2:** Summary of sequencing data after bisulfite transformation.

Groups	Samples	RL/bp	Discarded	CTS	CBS	BCR (%)
HYP	1HPP	150	2892	50,686,118	49,696,310	99.88
	2HPP	150	2680	49,831,685	48,750,517	99.88
	3HPP	150	2774	53,177,612	52,161,620	99.88
LYP	4LPP	150	2573	48,267,027	47,300,204	99.88
	5LPP	150	2792	51,087,490	49,943,458	99.88
	6LPP	150	3144	51,601,937	50,881,056	99.88

Note: 1HPP to 3HPP represent the samples from group HYP; 4LPP to 6LPP represent the samples from group LYP. RL: Read Length; CTS: Converted Top Strand; CBS: Converted Bottom Strand; BCR: Bisulfite conversion rate.

**Table 3:** Summary of the results of methylation site detection using Bismark.

Groups	Sample	TC	mCpG	mCHG	mCHH	mCU
HYP	1HPP	5,562,801,081	159,379,455	8,981,451	29,268,272	397,434
	2HPP	5,491,563,770	157,442,848	9,033,936	28,537,996	381,492
	3HPP	5,832,249,241	168,483,042	9,018,800	29,193,974	439,271
LYP	4LPP	5,332,035,985	155,724,744	8,731,969	27,987,834	424,279
	5LPP	5,646,859,847	165,634,409	9,845,431	30,559,020	401,850
	6LPP	5,711,482,994	169,359,413	9,461,311	30,459,101	343,792

Note: 1HPP to 3HPP represent the samples from group HYP, and 4LPP to 6LPP represent the samples from group LYP. mCpG: Total Methylated Cs in CpG context; mCHG: Total Methylated Cs in CHG context; mCHH: Total Methylated Cs in CHH context; mCU: Total Methylated Cs in Unknown context; mCpG: Percentage methylation (CpG context); mCHG: Percentage methylation (CHG context); mCHH: Percentage methylation (CHH context); TC: Total Cs analyzed.

## Data Availability

Data are contained within the article.
